# Is Hepatitis Delta infections important in Brazil?

**DOI:** 10.1186/s12879-016-1856-9

**Published:** 2016-09-29

**Authors:** Maira Ferreira Cicero, Nathalia Mantovani Pena, Luiz Claudio Santana, Rafael Arnold, Rafael Gonçalves Azevedo, Élcio de Souza Leal, Ricardo Sobhie Diaz, Shirley Vasconcelos Komninakis

**Affiliations:** 1 Retrovirology Laboratory, Federal University of São Paulo, 781 Pedro de Toledo Street, São Paulo, São Paulo Brazil; 2 Institute of Biological Sciences, Federal University of Pará, 01 Augusto Corrêa Street, Belém, Pará Brazil; 3 School of Medicine of ABC (FMABC), Clinical Immunology Laboratory, 821 Príncipe de Gales Avenue, Santo André, São Paulo Brazil

**Keywords:** Hepatitis Delta virus, Dried-blood spot, Founder effect, Phylogeography, Phylogenetic analysis, tMRCA, Brazil

## Abstract

**Background:**

The Hepatitis Delta Virus (HDV) can increase the incidence of fulminant hepatitis. For this infection occurs, the host must also be infected with Hepatitis B Virus. Previous studies demonstrated the endemicity and near exclusivity of this infection in the Amazon region, and as a consequence of the difficulty in accessing this area we used dried blood spots (DBS) in sample collection. The aims of this study were to investigate the presence of recombination, to analyze the epidemiology, ancestry and evolutionary pressures on HDV in Brazil.

**Methods:**

Blood samples from 50 individuals were collected using dried-blood spots (DBS 903, Whatman), and sent via regular mail to Retrovirology Laboratory from Federal University of São Paulo, where the samples were processed. In the analysis the following software were used: PhyML, RDP, BEAST, jModelTest and CODEML.

**Results:**

Our results confirm the prevalence of HDV-3 in the Amazon region of Brazil, with the absence of inter-genotypic recombination. It was identified a positive selection in probable epitopes of HDV on B lymphocytes that might indicate that the virus is changing to escape the humoral response of the host. The analysis of the time of the most common ancestor demonstrated the exponential growth of this virus in late 1970s that lasted until 1995, after which it remained constant. It was also observed a probable founder effect in two cities, which demonstrate the need to focus on prevention methods against HBV/HDV infection.

**Conclusion:**

We confirmed the prevalence of HDV-3 in the Amazon region of Brazil, without inter-genotypic recombination. The analysis of the time of the most common ancestor showed that this infection remain constant in the studied area. Taking into account the probable founder effect established in the cities of Rio Branco and Porto Velho, a focus on preventive methods is recommended against these infections.

## Background

Hepatitis delta is a disease that has been neglected by the health system [1], despite being responsible for the infection of 15 to 20 million people worldwide [2, 3]. In Brazil, 77 % of the infections with Hepatitis Delta Virus (HDV) occur in the North region.

The Northern region of Brazil has locations that are geographically isolated, which hinders the samples transportation until the processing laboratory. Thereat, it is used the dried blood spot (DBS) in sample collection at this region, for monitoring the HDV viral load (unpublished data). The use of DBS for sample collection and transportation is widely used in the HIV field, for monitoring the HIV viral load and the selection of drug resistance, on samples from remote sites or with limited financial resources. The results obtained using DBS sample collection are the same as using vacuum tubes [4–6].

HDV is the only subviral satellite agent known to infect humans. This virus requires the protection provided by the envelope of the Hepatitis B Virus (HBV) to enable its budding process from infected cells. Therefore, hepatitis delta occurs only in patients previously infected with HBV [7]. This co-infection increases the incidence of fulminant hepatitis is ten times [8–10], particularly when the characteristic Amazon genotypes (HDV-3 and HBV-F) are found combined [11, 12].

An important consideration regarding HDV is the availability of only one treatment option. This happens because this virus does not encode specific enzymes that could be used as target for the development of new antiviral drugs [3]. The efficacy of the treatment done with Interferon-Pegylated, is of only 43 %, and it produces very severe side effects [13].

Another situation that complicates the immune control of this infection is viral recombination. This event can allow the virus to escape the immune pressure imposed by the host's immune system, lead to an increase in viral fitness or even changes in the pathogenicity of the hybrid virus [14]. The recombination can occur when the individual is infected with a mixed viral population, as reported between genotypes 1 and 2 and between genotypes 1 and 4 in Taiwan [15, 16]. The probability of recombination occurring is greater when similar HDV genotypes cause the infection [17–19].

Perhaps because HDV-3 is the most distantly related among all HDVs genotypes, recombination with HDV-3 has never been reported [11, 19]. Furthermore, most studies with HDV-3 analyze only the coding portion of the genome and perhaps analyzing the entire genome, that is a much more detailed approach, may lead to different results in the identification of inter-genotypic recombination.

The adaptation of the virus to the environment can be measured by non synonymous (dN) and synonymous substitutions (dS) present in their genomes. The ratio between d*N*/d*S* is used to measure the strength and mode of natural selection acting on protein coding genes [20]. Results > 1 indicate that a protein is under positive selection, whereas ratio  < 1 denotes that the protein is evolving slowly under negative or purifying selection, and d*N*/d*S* ∼ 1 implies that the protein is evolving neutrally [21].

Another form to better understand an infection is to analyze its diversification, ancestry and phylogeography. These informations can be used to shed light on the demographic, social and biological factors that were responsible for the emergence and spread of the disease present in the population [22, 23]. This approach may also help in the development of preventive strategies against that specific pathogen.

Therefore, according to the above information, this study aims to investigate the presence of recombination and to analyze the epidemiology, ancestry and evolutionary pressures on HDV in samples collected with DBS from the Northern region of Brazil.

## Methods

### Samples and processing

A total of 50 samples from patients in medical care in different regional clinics at the Amazon region of Brazil was collected between 2009 and 2010. The samples were originated from the states of Acre (Cruzeiro do Sul and Rio Branco Cities), Amazonas (Manaus City) and Rondônia (Porto Velho City), all located in the North region of Brazil. The samples were collected and transported in dried-blood spots (DBS 903- Whatman). The mean age of the patients was 32 years (maximum of 69 and minimum of 18), and 52 % were male.

The samples were processed only after the CEP/UNIFESP (# 72971) was approved, and analyzed anonymously to ensure the protection of the participants. Informed consent was not required because the samples had been previously collected and stored. Patients co-infected with HIV and/or HCV were excluded.

The sample RNA was purified by the method developed by Boom [24], and the cDNA were synthesized using SuperScript III reverse transcriptase (Invitrogen, Carlsbad, CA). HDV full genome was sequenced using the shotgun technique, which amplified five overlapping fragments named A - E. The PCR methodology used was based on the study performed by Nakano [25]. The primers used amplified all HDV genotypes, and the sensitivity of amplification was up to 10 ^3^ copies/mL. For the D fragment a rescue strategy was necessary, where a nested PCR was implemented, combining the primers 1267_F with F2 [26] in the 1st round and A3 with 138_R in the 2nd round [26, 27].

The processing of all samples included a negative control to exclude the possibility of cross contamination and a positive control to ensure the reliability of the reaction. Moreover, both strands of the PCR were sequenced and thereafter a consensus sequence was created, which was used in the analysis to guarantee the best quality of the results obtained.

Sequencing was performed using the *ABI Prism 3130 Genetic Analyzer* (Applied Biosystems, USA). The sequences were edited using the *Sequencher* v.4.2 *Program* (Gene Code, Ann Arbor, USA) and analyzed using Blastn *http://blast.ncbi.nlm.nih.gov/Blast.cgi*), to verify their similarity with the database sequences.

### Sequence alignment and phylogenetic analyses

The HDV references sequences were obtained from the GenBank database (http://www.ncbi.nlm.nih.gov/genbank), described in Table 1. All the sequences were aligned using ClustalX software version 2.1 [28]. After the first alignment, the sequences were manually edited using the SE Al program version 2.0 (http://tree.bio.ed.ac.uk/software/seal/).

**Table 1:** Hepatitis Delta Virus sequences used as reference

Genotype	Origin	GenBank ID
1a	Iran	AY633627
	Taiwan	AF425644
1c	Somalia	U81988
	Ethiopia	HDU81989
2	Russia	AJ309879
	Japan	AB118846_M37
	Taiwan	X60193
3	Venezuela	AB037947, AB037949, AB037948
	Peru	L22063
4	Japan	AB118842
	Taiwan	AF209859
5	Togo	AM183326
	Senegal	AM183328
	Guinea-Bissau	AM183331
6	Nigeria	AM183329
	Cameroon	AJ584847
7	Cameroon	AM183333, AJ584844
8	Senegal	AM183327
	Congo	AJ584849

Maximum likelihood trees and bootstrap values were obtained using PhyML software [29]. The HKY model and gamma distribution (Γ) were selected and, based on the likelihood ratio test (LRT), implemented in the jModeltest software [30, 31]. The resulting trees were created and edited using FigTree (http://tree.bio.ed.ac.uk/software/figtree/).

### Recombination analyses

The RDP v.4 software was used to determine the presence of recombination in the sequences [32]. 3Seq performs an exact nonparametric test to detect recombination in triplets of sequences [33]. The maximum *χ*^2^ (MaxChi) is a method implemented by Maynard Smith [34], and it uses variable/invariable sites to detect recombination in pairs of sequences. The maximum match *χ*^2^ (Chimaera) is a modification of Smith’s method that uses variable sites to calculate the maximum *χ*^2^ match statistics [35]. Geneconv detects recombination by evaluating conserved substitutions in fragments between two sequences [36]. Although evolutionary methods are not explicitly implemented in Geneconv, it is robust and has low levels of false positive [37].

Initially, default parameters were used; then, these were optimized to avoid detection of false positive recombination. In addition, window sizes of 20 to 50 as well as Bonferroni correction were utilized.

### Phylogeographic analysis

A Bayesian Markov chain Monte Carlo (BMCMC) coalescent framework was used to estimate the ancestral genealogy, phylogeographic and time to the most common ancestor (tMRCA). After Markov chain runs enough so that the parameter space have been sufficiently visited, the mean and the 95 % HPD intervals of each parameter was calculated. The HKY model plus a gamma correction (Γ) was applied to all analyses; the evolutionary and demographic parameters were iteratively adjusted. We used the Bayesian discrete methods to estimate the node probability in regard to the geographical location of samples [38].

In order to explore the correlation between sample location and tree topology, the Bayesian discrete model [38] was used because it allows the reconstruction of ancestral location states while accounting for the uncertainties of maximum posteriori (MP) tree topology and mapping states at nodes.

The Bayesian skyline plot method (BSP) that provides an unbiased description of genetic diversity over time was used (expressed as the product of the effective sample size *Ne* and generation time τ). BSP contemplates a wide varied of demographic scenarios without formally assuming any determined model [39]. Since absolute time was used (years) to scale branch lengths and a specific generation time was not assumed, our estimates of *Ne.τ* will reflect only the relative genetic diversity of the infections over time.

The Markov Chain Monte Carlo (MCMC) processes were run for 3x10^8^ generations with the initial 10 % of each run discarded as burn in. The convergence of chains was evaluated using the TRACER software, version 1.5 (available at: http://beast.bio.ed.ac.uk/), runs were accepted when all parameters presented the effective sample size number (ESS) greater than 200. Two independent chains were run for each dataset and combined with LogCombiner software [39, 40].

The MP tree was inferred using the BSP method and the maximum clade credibility parameter were annotated using the TreeAnnotator software [39, 40]. All these analyses were performed with the BMCMC approaches implemented in the BEAST package version 1.8.0 [40].

### Estimates of the nonsynonymous (dN) and synonymous (dS) substitution rates

The codon based maximum likelihood method was used to estimate the selective pressures on the PTV sequences. This approach estimates the likelihood of distinct models of codon evolution and computes the ratio (*dN/dS* = ω) of the number of non-synonymous (*dN*) and synonymous (*dS*) substitution rates between sites considering the phylogenetic relationships of the sequences [41, 42]. To estimate the overall mean ratio, the one ratio model (M_0_) was used. This model assumes a single ω for all sites in the alignment and is the simplest model. The hypothesis of positive evolution is based on the likelihood ratio test (LRT) comparing pairs of nested models, with the corresponding statistics approximated by a chi square (*χ*^2^) distribution. To detect positive selection, we compared the null hypothesis of model M1, which allows for different proportions of conserved (ω = 0) and neutral codons (ω = 1), against the alternative hypothesis of model M2, which has an additional class of codons with ω ratio > 1, thus assuming positive selection. These models were implemented in the CODEML program of the PAML package, version 4.8, available at http://abacus.gene.ucl.ac.uk/software/paml.html.

## Results

### Phylogenetic overview

**Fig. 1:**
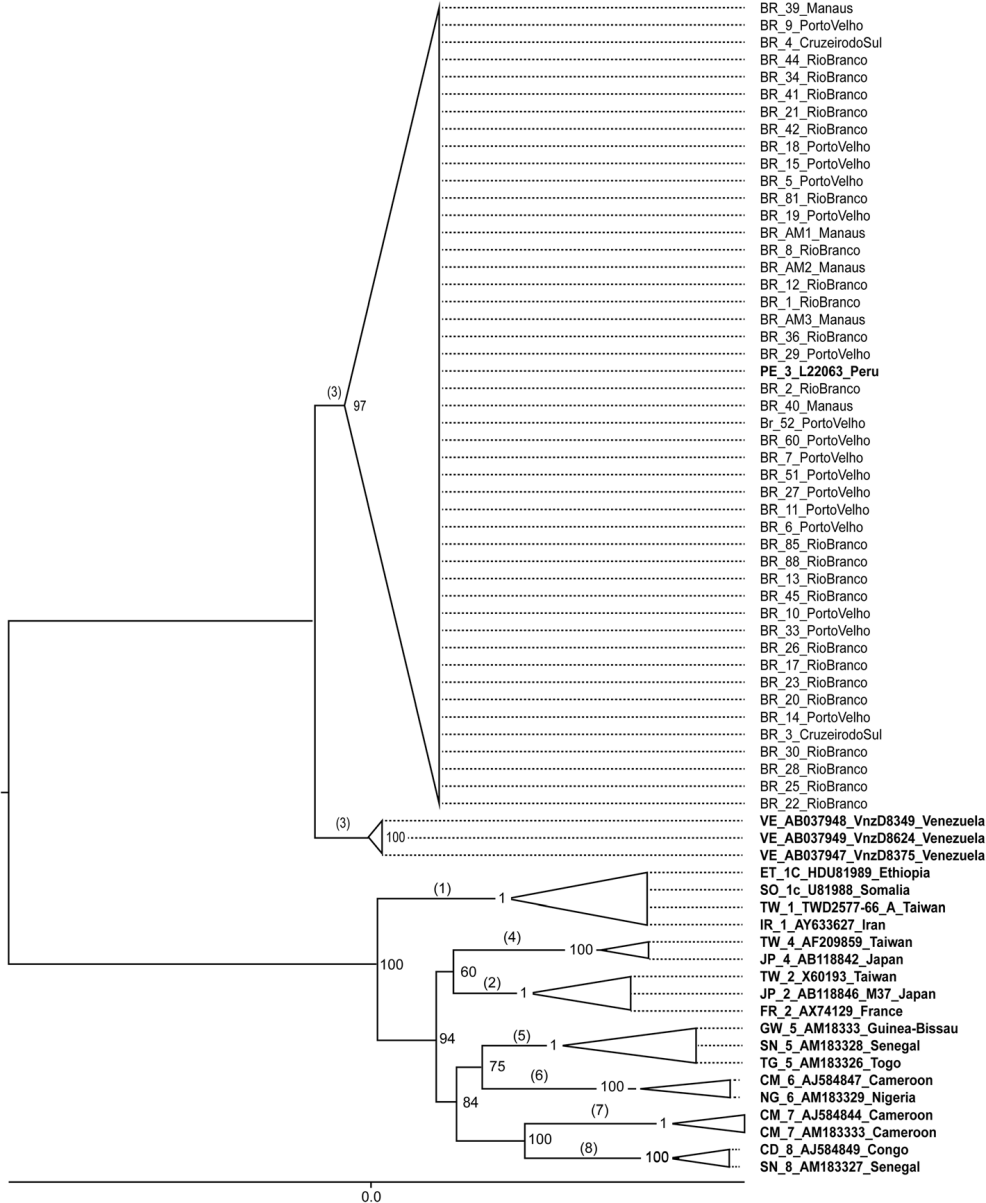
Phylogenetic analysis of the sequenced HDV genomes. Each triangle includes the sequences of a same genotype. The Maximum likelihood (ML) tree was constructed using the HKY + G + I model as implemented in PhyML software; values at the nodes of the tree are bootstrap support values obtained using 500 replicates. The tree was constructed using the genomes of 42 isolates generated in this study and the following reference genomes: genotype 1) IR_1_AY633627_Iran, TW_1_TWD2577-66_A_Taiwan, SO_1c_U81988_Somalia, ET_1C_HDU81989_Ethiopia; genotype 2) FR_2_AX74129_France, JP_2_AB118846_M37_Japan, TW_2_X60193_Taiwan; genotype 3) VE_AB037947_VnzD8375_Venezuela, VE_AB037949_VnzD8624_Venezuela, VE_AB037948_VnzD8349_Venezuela, PE_3_L22063_Peru; genotype 4) JP_4_AB118842_Japan, TW_4_AF209859_Taiwan; genotype 5) TG_5_AM183326_Togo, SN_5_AM183328_Senegal, GW_5_AM18333_Guinea-Bissau; genotype 6) NG_6_AM183329_Nigeria, CM_6_AJ584847_Cameroon; genotype 7) CM_7_AM183333_Cameroon, CM_7_AJ584844_Cameroon; genotype 8) SN_8_AM183327_Senegal, CD_8_AJ584849_Congo

Out of a total of 50 samples, 46 demonstrated the necessary quality for further analysis. The demographic information and the GenBank ID of each sample used in the analysis are detailed in Table 2 (present in the end of the manuscript). A maximum likelihood tree was initially constructed using the full length genomes of 46 samples and reference genomes of the main genotypes. Figure 1 shows that all sequences generated in the current study belong to genotype 3 and clustered within a single sequence with a bootstrap proportion of 97. Although the samples belong to genotype 3, they formed two groups: one composed of isolates from Yucpa Indians in Venezuela [43] and another composed of Brazilian isolates plus one isolate from the Yavari River in the Amazon basin close to the Peru Brazil border [19]. All sequences used to infer the tree mentioned above lacked a recombination signal.

**Table 2:** Demographic information and GenBank ID of the samples from our study

Origin	Sample ID	Age	Gender	GenBank ID
Rio Branco (23 samples)	1	36	Male	KF786351
	2^b^	-	Male	KF786323
	8	43	Male	KF786336
	12	58	Male	KF786343
	13	23	Female	KF786340
	17	48	Female	KF786308
	20	44	Female	KF786341
	21	-	Female	KF786348
	22	41	Male	KF786322
	23	23	Female	KF786334
	25	33	Male	KF786317
	26	39	Female	KF786339
	28	39	Male	KF786309
	30	-	Male	KF786316
	34	58	Male	KF786319
	36	36	Male	KF786324
	41	26	Female	KF786349
	42	23	Female	KF786335
	44	41	Female	KF786342
	45	39	Male	KF786350
	81	24	Female	KF786313
	85	41	Female	KF786330
	88	18	Female	KF786306
Cruzeiro do Sul (2 samples)	3	32	Male	KF786321
	4	42	Male	KF786346
Porto Velho (16 samples)	5	47	Male	KF786327
	6	24	Male	KF786328
	7^a^	-	Female	KF786331
	9	28	Male	KF786352
	10	31	Female	KF786307
	11	44	Male	KF786332
	14	26	Male	KF786337
	15	33	Male	KF786305
	18	38	Female	KF786325
	19	37	Male	KF786329
	27^a^	33	Female	KF786311
	29	50	Female	KF786345
	33	69	Female	KF786333
	51^a^	22	Female	KF786314
	52^a^	21	Male	KF786318
	60^a^	61	Male	KF786320
Manaus (5 samples)	AM1^b^	-	-	KF786310
	AM2^b^	-	-	KF786347
	AM3^b^	-	-	KF786338
	39	57	Male	KF786312
	40	34	Male	KF786326

^a^ samples from Porto Velho that are probably indigenous

^b^ samples excludes in further analysis

- samples without information

### Phylogeographic analysis of HDV-3 in the Amazon region

**Fig. 2:**
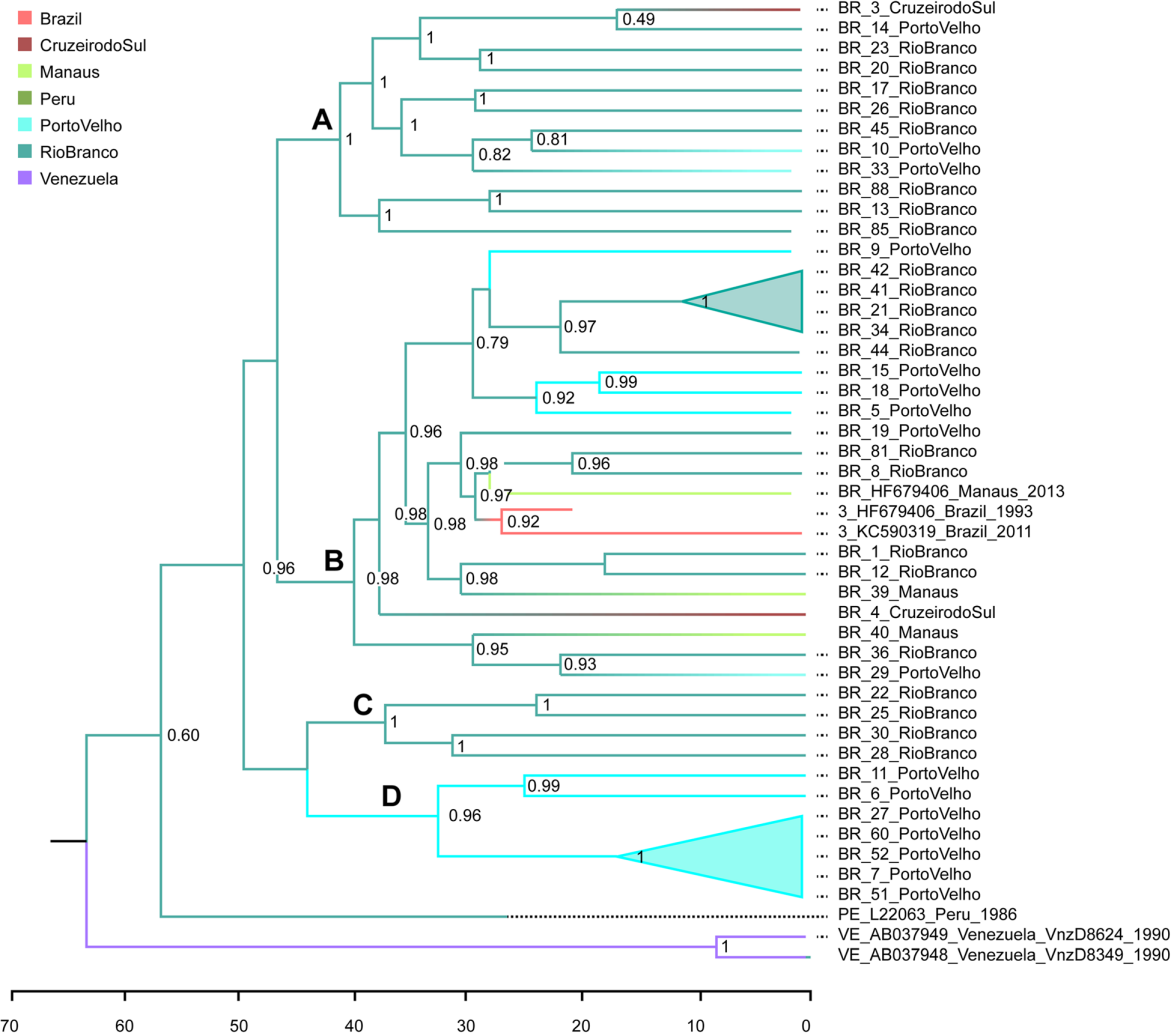
Phylogeography analysis of the HDV-3. The branch colors indicate the location from which samples were collected. The numbers at nodes indicate the location probability. The estimates were obtained using the full-length genomes of HDV-3 samples isolated from distinct locations in the Brazilian Amazon basin (i.e., Manaus, Porto Velho, Cruzeiro do Sul, Rio Branco). HDV-3 references from Venezuela and Peru were also used. The scale under the tree indicates the number of years before present time

42 samples were used for this analysis, four of the 46 original sequences were excluded because they had significant deletions along the genome. The phylogeography of HDV-3 isolates (Fig. 2), show that Brazilian samples clustered into four clades (named A, B, C, D), one sample from Peru isolated in 1986 is located at the base of these clades. The MP tree presents two large clusters, A and B, containing samples from different geographic locations (Rio Branco, Porto Velho, Cruzeiro do Sul and Manaus) and two small clusters, C and D, with sequences from Rio Branco and Porto Velho, respectively.

The topology also indicates two sets of nearly identical isolates: one from Rio Branco and another from Porto Velho (filled triangles in Fig. 2). The branching pattern of intermingled samples from Rio Branco and Porto Velho suggests a bidirectional flow of infections between these two locations.

The pattern of geographical dispersal of HDV-3 is depicted by the highest location probability on the nodes of the MP tree (Fig. 2; the location is indicated by colored branches and the location probability by numbers at the nodes). Although the multi-location clades A and B indicate that Rio Branco contributes significantly to the geographical spread of HDV-3, this may occur because samples from this location were overrepresented. When random sets of sequences containing equivalent numbers of samples from Rio Branco and Porto Velho were evaluated, the location probability to support Rio Branco at internal nodes decreased to values below 0.7 (*data not shown*).

### The population dynamics of HDV-3

**Fig. 3:**
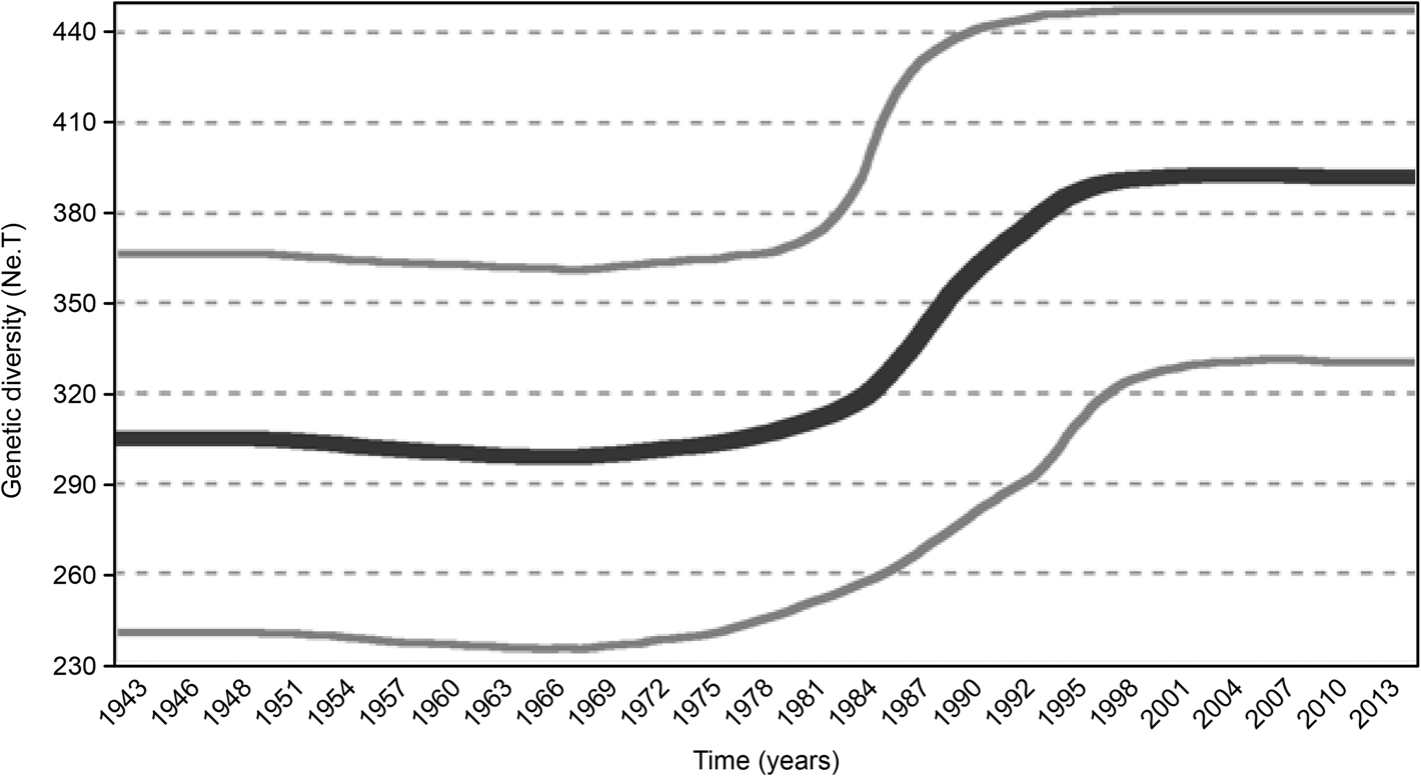
Bayesian skyline plot (BSP) of the population dynamics of HDV-3. The plot represents the genetic diversity (y-axis) against chronological time expressed in years (x-axis). The thick line indicates the mean and the thin lines the 95 % confidence posterior interval of the Ne.τ. The estimates were obtained using the full-length genomes of 42 HDV-3 isolates

Figure 3 shows the reconstructed dynamics of the genetic diversity (*Ne*.τ, y axis) plotted against time in years (x axis). The plot shows the mean (thick solid line) and the 95 % highest density interval (thin solid lines). The shape of the plot demonstrates a period of exponential growth that began in the late 1970s and lasted until 1995. The estimated tMRCA values for the HDV-3 and for the Amazon clade were 1934 (1927 until 1940) and 1952 (1948 until 1955), respectively.

### Selective regimen in the large HDV antigen (L-HDAg)

A systematic codon based analysis was performed on HDV isolates using the 215 codons from the L-HDAg region. In Table 3, the estimates of the codon based analysis are summarized. To detect positive selection models M7 and M8 were compared using likelihood ratio test assuming two degrees of freedom between models. Initially an intra-subtype analysis was performed using a dataset with 47 isolates of HDV-3, 46 from this study and one reference sequence from Peru (L22063), results of Model M8 indicated 24 codons in the L-HDAg under positive selection with mean ω values of 5.04.

**Table 3:** Codon-based analysis of d_N_/d_S_ rates in the L-HDAg gene of Hepatitis Delta virus

Model	*p*	-nlL	Estimated parameters	Sites^b^
Site-specific models for HDV-3 (*n* = 47)				
M7 (beta)	2	5471.30397	*p* = 0.09695 *q* = 0.15394 k (ts/tv) = 3.53356	Not allowed
M8 (beta&ω)^a^	4	5293.55296	*p*_*0*_ = 0.82801 *p* = 0.11508 *q* = 0.24197 (*p*_*1*_ = 0.17199) ω = 5.03937 k (ts/tv) = 4.64237	4P, 8 K, 9 K, 29E, 37 T, 44 L, 54I, 55 V, 63D, 73P, 77A, 80I, 82S, 84P, 96Q, 120S, 123Q, 130G, 134 K, 143 V, 169 N, 190 T, 198 F, 205H
Site-specific models for the others HDV Genotypes (*n* = 78)				
M7 (beta)	2	9893.49320	*p* = 0.27034 *q* = 0.48352 k (ts/tv) = 2.60287	Not allowed
M8 (beta&ω)^a^	4	9781.56864	*p*_*0*_ = 0.92957 *p* = 0.31395 *q* = 0.63880 (*p*_*1*_ = 0.07043) ω = 3.27612 k (ts/tv) = 2.85894	6 L, 8 K, 9 K, 16I, 37 T, 120S, 157G, 179A, 187I

*InL* Maximun likelihood of each model

^a^ Best-fit model according to the LRT comparing the M7 and M8 models, using two degrees of freedom and a significance level of 0.010

^b^ Positive selected codons were identified according to the Bayes Empirical Bayes (BEB) analysis with *P* > 99 %

When this same analysis was performed with other HDV genotypes (78 sequences) the Model M8 indicated only nine codons under positive selection, four of which were similar with the result demonstrated with the HDV-3 genotype (8 K, 9 K, 37 T and 120S).

## Discussion

Chronic HDV/HBV infection is perhaps the most intriguing and difficult to treat among the human viral hepatitis and is one of the most neglected diseases worldwide [44]. The study area included was based upon previous articles that demonstrated the endemicity and near exclusivity of HDV infection in the Amazon region of Brazil [12, 19, 45, 46], only two studies have identified HDV cases outside the locations presented in this study [47, 48].

In Brazil, 77 % of the HDV infections occur in the North region [48]. The isolation of this virus in this location can be a consequence that the majority of HDV cases are present in the indigenous population, that generally lives in isolated regions and interacts preferentially among individuals of their own tribes.

The DBS was used to collect the samples because: distant areas were included, this method is less invasive for the patient once the blood is collected via puncture of the finger using a lancet, it requires minimal collector training, allows the samples to be stored at room temperature with transportation in envelopes sent via regular mail (less expensive than using dried ice transportation) [49, 50].

Based upon the phylogenetic tree created from the sequences obtained in this study, it was possible to conclude that all samples belongs to genotype 3, being clustered (97 in the bootstrap) along with one HDV-3 sequence from Peru. These results confirm previous studies, that also demonstrated the high prevalence of this genotype in the studied area [12, 19, 45, 46].

It was not detected any inter-genotypic recombination. This may have happened because we analyzed areas of difficult access, which may have hindered the introduction of different HDV genotypes.

The BMCMC method was used in the phylogeographic analysis because it incorporates the uncertainty of the measurements by considering the intrinsic errors both in the tree reconstruction and in the coalescent method. These considerations guarantee more confiability in the results obtained. This analysis demonstrated the presence of four clades in HDV-3 samples from the Amazon region (A, B, C and D). Two of them (A and B) contained samples from all the locations studied (Rio Branco, Porto Velho, Cruzeiro do Sul and Manaus), whereas clusters C and D were significantly smaller and represented the cities of Rio Branco and Porto Velho, respectively. This analysis also indicates two clusters with nearly identical sequences, one from Rio Branco (composed of four samples) and another from Porto Velho (composed of five samples), which suggests a bidirectional flow of infection in these two cities.

Rio Branco is the capital city of the Acre State, populated in the beginning of the 20th century by natives who already lived in the area, along with the migration of people from Northeast of Brazil and immigrants from Turkey, Portugal and Lebanon attracted by the enrichment of the rubber era [51]. The cluster identified at this location indicates a probable founder effect, that may suggest that the dissemination of this infection was introduced by an indian (HDV-3 is characteristic of the indigenous population), and not brought with the city foundation.

Porto Velho, the capital city of Rondônia State, was founded as a result of the railroad “Madeira Mamoré”, widely used in the rubber and gold eras. Nowadays several indigenous tribes still live in this region [51]. Five samples from this location were probably indigenous (surnames characteristic from tribes living in this area), and they grouped together into a single cluster. This situation demonstrates that probably a single virus caused the spread of the HDV infection in this region, as well.

These probable founder effects demonstrate the necessity of focusing on prevention strategies against HBV and HDV infection, mainly in the North of Brazil. This region represents respectively 14 and 77 % of the total cases of these two viral infections. The most effective prevention against HDV is the HBV vaccination, since the HDV depends of the HBV for its budding from the infected cell, and if HDV is not contained it can be disseminated to other regions of Brazil, causing a public health problem.

The analysis of the most common ancestor time (tMRCA) showed that the infection of HDV experienced an exponential growth period in the late 1970s that lasted until 1995 and has been maintained since then. In 1989, the HBV vaccination was implemented in the Amazon region and in 1993, the minimal age for vaccination was decreased, these events could explains the plateau in the exponential growth of HDV after 1995; but this same plateau does not exclude the need to focus on preventive campaigns against this infection, since its treatment is not effective, no remission has been observed and the vaccination against HBV is not effective in all vaccinated individuals.

It was also observed that the HDV-3 has more codons under positive selection in the L-HDAg than the other HDV genotypes, but four codons (8 K, 9 K, 37 T and 120S) were present in both analysis indicating that these modification are not genotype specific. Some modifications were located in probable epitopes of HDV for B lymphocytes (amino acids 3–18; 107–122 and 170–196) [52, 53]. This finding may indicate that the virus is evolving to escape the humoral response of the host.

The variation of epitopes at amino acids 174 to 195, often emerges after severe hepatitis attacks during the chronic phase of infections [15]. Changes in epitopes are common during the chronic phase of infections because of the intense selection forces by the immune defense against the virus [54]. Unfortunately, we did not have information to confirm that these individuals were in this phase of infection.

## Conclusion

Our results confirm the prevalence of HDV-3 in the Amazon region of Brazil. This geographically isolated region may have hindered the introduction of different HDV genotypes, which may account for the absence of inter-genotypic recombination. The analysis of the most common ancestor time showed that the infection with HDV experienced an exponential growth period in the late 1970s that lasted until 1995, after which it has remained constant. Taking into account the probable founder effect established in the cities of Rio Branco and Porto Velho, a focus on preventive methods is recommended against this infection, particularly in the North of Brazil. A positive selection was also found in probable epitopes of HDV for B lymphocytes, which may indicate that the virus is evolving to escape the humoral response of the host.

## Abbreviations

BMCMC: Bayesian Markov chain Monte Carlo
BSP: Bayesian skyline plot method
cDNA: Complementary DNA
DBS: Dried blood spots
dN: Non synonymous substitutions
dS: Synonymous substitutions
ESS: Effective sample size number
HBV: Hepatitis B Virus
HDV: Hepatitis Delta Virus
HPD: Highest posterior density
L-HDAg: Large HDV antigen region protein
LRT: Likelihood ratio test
MP: Maximum posteriori
PCR: Polymerase chain reaction
tMRCA: Time to the most common ancestor
